# Cardiac Adrenergic Activation in Heart Failure With Preserved Ejection Fraction: A Role for β-Blockade?∗

**DOI:** 10.1016/j.jacbts.2022.01.003

**Published:** 2022-02-28

**Authors:** Michael R. Bristow, Douglas L. Mann

**Affiliations:** a Cardiology Division, Department of Medicine, University of Colorado, Aurora, Colorado, USA; b ARCA Biopharma, Inc, Westminster, Colorado, USA; c Center for Cardiovascular Research, Washington University School of Medicine, St. Louis, Missouri, USA

**Keywords:** adrenergic nervous system, cytokines, HFpEF, transcardiac sampling

**Figure:**
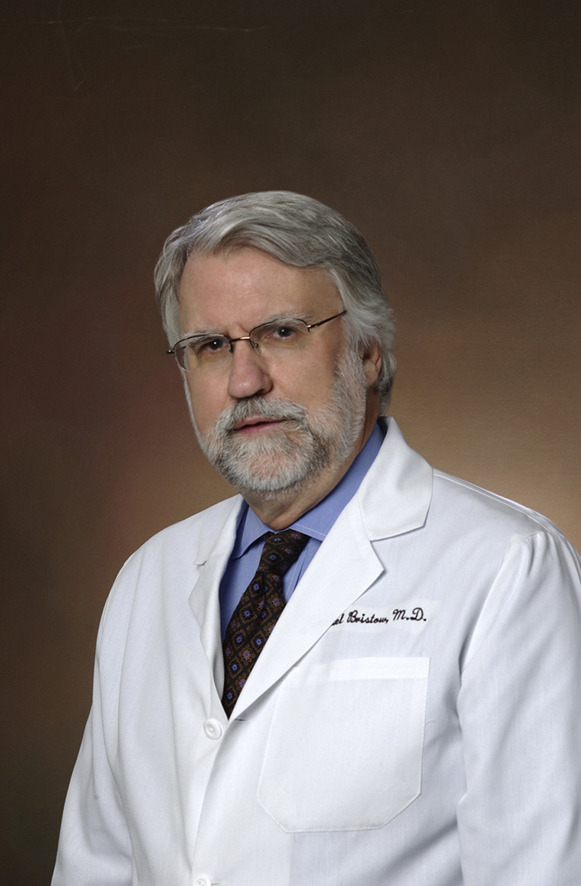

The recent clinical success with sodium-glucose cotransporter 2 (SGLT2) inhibitors and angiotensin receptor–neprilysin inhibitors (ARNIs) in treating heart failure patients with reduced ejection fraction (HFrEF) (left ventricular ejection fraction [LVEF]: ≤40%) and preserved ejection fraction (HFpEF) (LVEF: ≥50%) has given rise to the proposition that LVEF cutoffs should no longer be used as the basis for subclassifying heart failure patients into different pathophysiologic phenotypes and that, going forward, for classification systems to have clinical importance, they should be “more representative of the state of our knowledge about heart failure, and should reflect contemporary approaches to how patients with heart failure are evaluated and treated.”^1^ With this brief context as background, the results by Kaye et al,^2^ in this issue of *JACC: Basic to Translational Science* raise provocative questions about the state of our knowledge about heart failure, as well as where and how the field should advance.^2^

Kaye et al^2^ measured systemic arterial and coronary sinus blood in 20 heart failure patients with an LVEF of >50% compared to and 14 healthy volunteers to investigate the relative activities of the adrenergic (sympathetic) nervous system and inflammatory pathways. Importantly, patients with coronary artery disease, valvular heart disease, and significant pulmonary disease were excluded. The diagnosis of HFpEF was confirmed by the presence of a pulmonary capillary wedge pressure of ≥15 mm Hg at rest or ≥25 mm Hg during symptom-limited exercise. Arterial and coronary sinus plasma catecholamine concentrations were evaluated by high-performance liquid chromatography. Plasma arterial and coronary sinus concentrations for 92 inflammatory proteins were determined using proximity extension assay technology (Olink Proteomics). The major findings of the study by Kaye et al^2^ were of a significant increase in plasma arterial levels of norepinephrine (NE) and dihydroxyphenylglycol (DHPG), a major intraneuronal metabolite of NE reuptake, in HFpEF patients as compared to healthy subjects. Kaye et al^2^ found that the trans-cardiac concentration gradient for NE was significantly increased in HFpEF patients, whereas there was a trend toward increased DHPG release, suggesting no major effect on neuronal reuptake of NE. Interestingly, HFpEF patients in atrial fibrillation had significantly elevated arterial NE levels and increased transcardiac NE concentrations in comparison to patients in sinus rhythm. Although a principal component analysis revealed that the expression pattern of inflammatory biomarkers was significantly different in the arterial blood samples from HFpEF patients as compared to healthy subjects, the transcardiac gradient of the inflammatory biomarkers did not differ between healthy subjects and HFpEF patients. Kaye et al^2^ concluded that HFpEF is characterized by both cardiac and systemic adrenergic activation, whereas there is peripheral inflammation in HFpEF but no detectable myocardial release of inflammatory cytokines.

The paper by Kaye et al^2^ adds much needed data to the characterization of the HFpEF heart failure phenotype. Adrenergic mechanisms in HFrEF have been extensively studied and reported, with much of the work done by this group.^3^ In HFrEF, cardiac adrenergic activation is pronounced and provides an important component to the pathophysiologic rationale for β blocker therapy. In contrast, there have been no previous reports of the status of cardiac adrenergic mechanisms in human HFpEF. However, work in animal models,^4^ systemic NE measurements in HFpEF subjects,^5^ and limited data on the response to β_1_-adrenergic receptor blockade in heart failure patients with LVEFs of >50%^6^ suggest that cardiac adrenergic activation occurs in HFpEF.

Based on elevated transcardiac levels, Kaye et al^2^ have convincingly demonstrated that cardiac adrenergic activation is indeed present in HFpEF. Increased neuronal release vs impaired reuptake appeared to be the dominant mechanism, inasmuch as the transcardiac gradient of DHPG was not significantly increased. In terms of factors that may have led to cardiac adrenergic activation, increased left atrial filling pressures were moderately positively correlated with transcardiac NE concentrations, similar to what has been reported in HFrEF. Arterial NE plasma concentrations were also elevated in HFpEF, in agreement with previous reports of elevated systemic levels,^5^ and it is likely that a substantial component of the generalized circulatory increased NE had a cardiac origin.

A second interesting aspect of the paper by Kaye et al^2^ relates to the observation that there was no detectable myocardial release of inflammatory cytokines, despite evidence of peripheral inflammation in the HFpEF patients. These observations differ somewhat from those reported in HFrEF patients, in whom transcardiac gradients for inflammatory mediators have been reported in some,^7^ but not all, studies.^8^ It should be recognized, however, that because of the differences in the plasma half-life of NE, which is on the order of minutes, vs inflammatory mediators, whose half-lives in the circulation can be prolonged by binding to cognate circulating receptors that have been shed (released), the kinetics of peripheral degradation of NE may favor the detection of transcardiac gradients for NE far more readily than the detection of a transcardiac gradient for inflammatory mediators. That is, the signal of NE release may be easier to measure against the lower background noise of low NE levels secondary to rapid degradation, whereas the signal of inflammatory peptide release from the heart may be more difficult to measure against the higher background noise of elevated levels of circulating inflammatory peptides that are removed from the circulation more slowly.

Do the findings by Kaye et al^2^ bring the field closer to a unified approach to treating all heart failure patients with the same medications? Prospective randomized trials have clearly established the effectiveness of SGLT2 inhibitors and ARNIs in reducing heart failure hospitalizations in patients with HFrEF and HFpEF.^1^ Moreover, the benefit of aldosterone receptor antagonists on heart failure outcomes has been established prospectively in randomized trials in HFrEF patients and in a subgroup analysis of a prospective randomized clinical trial of HFpEF patients.^9^ Although β-blockers are effective in reducing cardiovascular death and heart failure hospitalizations in HFrEF patients, β-blockers have not been evaluated prospectively in large randomized clinical trials in HFpEF patients, in part because of the concerns over worsening exercise capacity secondary to chronotropic incompetence, which may affect approximately 30% to 50% of patients with HFpEF.^10^ The obvious question raised by the studies by Kaye et al is whether antiadrenergic therapy would be effective, particularly in subjects with higher NE elevations. In the SENIORS (Study of Effects of Nebivolol Intervention on Outcomes and Rehospitalization in Seniors With Heart Failure) trial, in whom 15% of the patients had an LVEF of >50%, the numerically lower HR and overlapping CIs in subjects with LVEFs of >46% (0.76; 95% CI: 0.52-1.11) as compared to the overall SENIORS cohort (0.86; 95% CI: 0.72-1.04)^6^ suggests that at least some HFpEF patients, perhaps those patients with higher levels of adrenergic activity, might benefit from β-blocker therapy. The exciting findings by Kaye et al in this issue of *JACC: Basic to Translational Science* raise the intriguing possibility that it may be possible to identify such hyperadrenergic subphenotypes of HFpEF patients in whom β blockers may prove to be effective in reducing heart failure events and suggest that this is an area that needs further investigation. However, at the time of this writing, the currently available limited clinical trial data for β-blockade in HFpEF, defined as heart failure with an LVEF of >50%, do not currently support the general application of β blockers in this setting.

## Funding Support and Author Disclosures

Dr Bristow is an officer and director of ARCA Biopharma, which is developing a pharmacogenetically targeted β-blocker for treatment of atrial fibrillation in heart failure patients. Dr Mann has reported that he has no relationships relevant to the contents of this paper to disclose.

## References

[1] Greenberg B., O’Connor C.M., Felker G.M. (2021). Classifying heart failure in the 21st century: matching taxonomy with science. J Am Coll Cardiol HF.

[2] Kaye D.M., Nayakkara S., Wang B. (2022). Characterization of cardiac sympathetic nervous system and inflammatory activation in HFpEF patients. J Am Coll Cardiol Basic Trans Science.

[3] Eisenhofer G., Friberg P., Rundqvist B. (1996). Cardiac sympathetic nerve function in congestive heart failure. Circulation.

[4] Jacobsen M.D., Weil M., Raff M.C. (1996). Role of Ced-3/ICE-family proteases in staurosporine-induced programmed cell death. J Cell Biol.

[5] Kitzman D.W., Little W.C., Brubaker P.H. (2002). Pathophysiological characterization of isolated diastolic heart failure in comparison to systolic heart failure. JAMA.

[6] van Veldhuisen D.J., Cohen-Solal A., Bohm M. (2009). Beta-blockade with nebivolol in elderly heart failure patients with impaired and preserved left ventricular ejection fraction: data from SENIORS (Study of Effects of Nebivolol Intervention on Outcomes and Rehospitalization in Seniors With Heart Failure). J Am Coll Cardiol.

[7] Tsutamoto T., Wada A., Matsumoto T. (2001). Relationship between tumor necrosis factor-alpha production and oxidative stress in the failing hearts of patients with dilated cardiomyopathy. J Am Coll Cardiol.

[8] Grossman G.B., Rohde L.E., Clausell N. (2001). Evidence for increased peripheral production of tumor necrosis factor-alpha in advanced congestive heart failure. Am J Cardiol.

[9] Pfeffer M.A., Claggett B., Assmann S.F. (2015). Regional variation in patients and outcomes in the Treatment of Preserved Cardiac Function Heart Failure With an Aldosterone Antagonist (TOPCAT) trial. Circulation.

[10] Borlaug B.A., Olson T.P., Lam C.S.P. (2010). Global cardiovascular reserve dysfunction in heart failure with preserved ejection fraction. J Am Coll Cardiol.

